# Physical Activity Levels of Adolescents and Adults With Cerebral Palsy in Urban South Africa

**DOI:** 10.3389/fneur.2021.747361

**Published:** 2021-10-28

**Authors:** Roshaan Salie, Maaike M. Eken, Kirsten A. Donald, A. Graham Fieggen, Nelleke G. Langerak

**Affiliations:** ^1^Neuroscience Institute, Faculty of Health Sciences, University of Cape Town, Cape Town, South Africa; ^2^Division of Neurosurgery, Faculty of Health Sciences, University of Cape Town, Cape Town, South Africa; ^3^Institute of Sport and Exercise Medicine, Division of Orthopaedic Surgery, Department of Surgical Sciences, Faculty of Medicine and Health Sciences, Stellenbosch University, Cape Town, South Africa; ^4^Department of Paediatrics and Child Health, Red Cross War Memorial Children's Hospital, University of Cape Town, Cape Town, South Africa

**Keywords:** cerebral palsy, lifespan, accelerometry, GMFCS, low-to-middle income country

## Abstract

**Background:** Research in high income countries shows that people with cerebral palsy (CP) are less physically active than typically developing (TD) peers, but less is known regarding physical activity (PA) in those with CP in low-to-middle income countries. The aim of this study was to determine daily step count and levels of PA in adolescents and adults with CP living in urban South Africa, compared to TD peers, and to determine associations with sex, Gross Motor Function Classification System (GMFCS) level, body mass index and socio-economic status.

**Materials and Methods:** This case–control study included 26 adolescents and 22 adults with CP (GMFCS Level I-V) and matched TD peers (25 and 30, respectively). Participants wore an ActiGraph GT3X for 7 consecutive days to determine step count and time (minutes per hour) spent in PA levels, including sedentary (SED), low physical activity (LPA) and moderate to vigorous physical activity (MVPA).

**Results:** The daily step count and PA levels for ambulant adolescents with CP (GMFCS level I-III) were similar to TD peers, while this was less for adolescents classified in GMFCS level IV-V. Daily step count, SED and MVPA were similar for adults classified in GMFCS level I-II compared to TD adults, while all parameters were lower for adults using assistive devices (GMFCS level III) and non-ambulant adults (GMFCS level IV-V) compared to TD peers. Daily step count and PA levels were inversely associated with GMFCS, while no other associations were found.

**Conclusion:** People with CP who were more mobile dependent (higher GMFCS level) were less active. However, adolescents and adults with CP classified as GMFCS levels I-II living in urban South Africa recorded similar step count and PA levels as their TD peers. This was also the case for adolescents using assistive devices, though not for those in the adult group (GMFCS level III). Furthermore, it was apparent that even the ambulant individuals with CP and TD cohorts were relatively inactive. Intervention programs for CP and TD adolescents should be aimed at finding strategies to keep adolescents physically active well into adulthood, in order to promote physical health, social and emotional well-being and independence.

## Introduction

Cerebral palsy (CP) is the one of the most common motor disabilities in children (1), with a reported prevalence of 2–3 cases per 1,000 live births. However, these figures are largely drawn from studies in high income countries (HIC). The prevalence is estimated to be as high as 10 cases per 1,000 in low-to-middle income countries (LMIC) (2). According to the World Health Organization (3), the number of adolescents is expected to increase through to 2050, especially in LMIC where close to 90% of the 10–19 year old population live. This, together with improved survival of children with CP into adulthood (1), highlights the importance of preparing the healthcare system to address important challenges faced by people with CP.

It is widely understood that CP is a permanent disorder of posture and movement, attributed to a non-progressive disturbance that occurred in the developing fetal or infant brain (4). Motor disturbances in CP such as impaired motor planning, coordination, motor learning and fine motor skills may lead to the limitation of activities and activity levels starting before adulthood (5). Evidence suggests that developing healthy patterns of physical activity (PA) as an adolescent with CP greatly increases the probability of maintaining healthy PA levels as an adult (6). Exercise, however, is often poorly utilized in populations with CP (7), despite the positive effects it has on physical function (8).

Compared to the typically developing (TD) general population, individuals with CP are frequently reported to have reduced physical fitness levels (7) and to be less physically active (9, 10). Reasons for this inactivity could be due to different factors like physical impairments, but probably the lack of knowledge and life skills, limited availability of transport and professional guidance as well as social support plays a role (11). McKenzie et al. (12) concluded in a systematic review that (young) adults struggle to find the right balance to be physically active because of their social and physical environment. However, since these studies are mainly focused on HIC, little is known about the influence of cultural and socio-economic factors on the level of an individual's PA in resource-limited settings such as South Africa.

A recent systematic review by Malek and colleagues (13) regarding research on CP in LMICs, reported that research studies focused on the impairments of individuals with CP, rather than their functioning. In other words, the literature mainly comprises research addressing issues of body structure and function as conceptualized in the International Classification of Functioning, Disability and Health (ICF) (14) framework, while there is a lack of published work with a focus on the level of activities, participation and contextual factors. These authors also acknowledged that the research in Africa is mainly focused on children with CP, with few studies focusing on adolescents and adults with CP (13).

Very little has been reported on PA levels in adolescents and adults with CP or TD peer groups living in LMIC settings. Based on the knowledge gained from studies conducted in HIC and recognizing that there might be additional barriers in LMIC, it is hypothesized that adolescents and adults with CP will be less active than their TD peers. Understanding the complete picture, particularly regarding levels of PA in individuals with CP, may support strategies to address the possible challenges faced during transition as well as to promote healthy aging for individuals with CP living in LMIC. Therefore, the aim of this study was to determine the levels of PA in adolescents and adults with CP living in urban South Africa, compared to TD peers, and to determine whether there are associations between PA levels and contextual factors sex, Gross Motor Function Classification System (GMFCS) level, Body Mass Index (BMI) and socio-economic status (SES).

## Methods

### Study Design and Participants

This case-control study is a sub-study of a bigger research project (15) focusing on the challenges, needs and well-being of adolescents and adults with CP (GMFCS Levels I-V) and TD peers (matched for sex, age and SES) living in urban South Africa. The cohorts were based on a convenience sample, where the adolescents with CP were recruited from three different schools for Learners with Special Education Needs (LSEN) in Cape Town South Africa, where they were in one of the last 3 years of school (grades 10–12). The TD adolescents were recruited from a mainstream school in Cape Town, and matched for age, sex and SES. Adults with CP were recruited from databases of previous studies (16, 17), referrals, word of mouth suggestions and social media. They were included if they had a medical diagnosis of CP, had attended a special school at least 5 years prior to the study, and were aged between 23 and 40 years. Adults in the TD cohort were similarly matched for age, sex, and SES to the adults with CP. Adolescent and adult participants were excluded if they had any neuromuscular or physical disorders unrelated to CP, and if they were unable to effectively understand and communicate in English or Afrikaans.

Each adolescent and adult who showed interest in participating in the study and met the selection criteria was contacted to make an appointment. During this meeting the procedure for the study was explained, the written informed consent form was signed by the participants (and when appropriate their caregivers), instructions were given on how to use the Actigraph and a date was set to collect the device after testing.

This study was approved by the Human Research Ethics Committee at the University of Cape Town, South Africa. Permission was also granted by the Western Cape Education Department and as well as by the principals of the three LSEN schools. The study was conducted in line with the principles set out in the Declaration of Helsinki (18).

### Background Information

Participant's characteristics including age, sex, CP subtype, and SES were obtained. The latter was estimated based on housing density, which was calculated by dividing the number of people living in the house by number of rooms within the house (excluding kitchen and bathroom). The housing density was then categorized as low SES (housing density score >1.5), average SES (score 1–1.5) and high SES (score <1) (19). In addition, height and weight were measured to determine BMI, which was then categorized into underweight (BMI <18.5), healthy (BMI 18.5–24.9), overweight (BMI 25–29.9) and obese (BMI > 30) (20). Adolescents and adults with CP were also classified for gross motor function with the GMFCS (21), manual ability according to the Manual Ability Classification System level (MACS) (22), as well as the participant's ability to communicate using the Communication Function Classification System (CFCS) (23).

### Accelerometry

The tri-axial ActiGraph GT3X accelerometer (ActiGraph, Shalimar, FL, USA; mass: 19 g, dimensions: 4.6 x 3.3 x 1.5 cm) was used for this study, which is a waist-worn accelerometer used to objectively measure the daily step count and levels of PA. In this manner, PA was divided into time spent sedentary (SED), in low physical activity (LPA), moderate physical activity (MPA) and vigorous physical activity (VPA). Different cut-points [counts per minute (cpm)] have been validated (24) but for the current study cut-points defined by Freedson (25) (developed for adults) and Evenson (26) (developed for children) were chosen. Therefore, the following cut-points were utilized: SED: Freedson 0 to 99 cpm, Evenson 0 to 100 cpm; LPA: Freedson 100 to 1,951 cpm, Evenson 101 to 2,295 cpm; MPA: Freedson 1,952 to 5,724 cpm, Evenson 2,296 to 4,011 cpm; and VPA: Freedson > 5,725 cpm, Evenson > 4,012 cpm. The ActiGraph has been used in individuals with CP as well as the TD population (27–31), and research showed evidence to support its reliability (32) and validity in children (30, 31, 33) adolescents (30, 32) and adults with CP (27, 28).

### Data Collection and Analyses

The ActiGraph was placed on the participant's non-dominant hip (27, 28, 34) and secured around the waist. The accelerometer was set and initialized to record magnitudes of movement triaxially in epochs of 5 s with a sampling frequency of 30 Hz (default setting). Participants were asked to wear the ActiGraph consecutively for 7 days, and only to removed it prior to bathing/showering or swimming.

The ActiLife software (Pensacola, FL, USA) was used to process the recordings and transform epoch lengths to 60 s (35). Sleep time was set at non-wear time. Valid wear time per day was determined by using similar protocols as also applied in other studies with adolescents (31) as well as adults (27, 36). First a valid day was set at a minimum of 5 h per day (300 min), excluding sleep time. Participant's data were considered for further analysis when a minimum of 3 valid weekdays were recorded and a minimum of 1 valid weekend day. The valid days of wear time did not have to be consecutive.

For each participant, an average of daily step count and SED, LPA and MVPA (MPA plus VPA) were determined. Thereafter step count and PA data was reported for weekdays and weekend days together (“whole week”) as well as separately (“weekdays” and “weekend days”).

### Statistical Analysis

Shapiro-Wilk tests were conducted to determine the normality of the data. This resulted in the use of non-parametric statistical analyses for the description of the personal and environmental factors, as well as daily step count and PA data (time spent in SED, LPA and MVPA) for the adolescents and adults with CP and matched TD cohorts. Based on GMFCS, adolescents and adults with CP were subdivided into 3 groups including: CP I-II: independently ambulant and classified as GMFCS levels I and II; CP III: required assistive devices for ambulation and classified as GMFCS level III; and CP IV-V: non-ambulant and classified in GMFCS levels IV and V.

Kruskal-Wallis tests were used to determine differences in daily step count and PA data between the CP and TD groups. If Kruskal-Wallis showed a significant group effect, post-*hoc* testing was performed to analyse the differences between the 4 groups (i.e., CP I-II, CP III, CP IV-V, and TD). Spearman rho correlations were calculated to determine any associations between PA data with sex, GMFCS, BMI and SES. A Bonferroni correction was applied to correct for multiple analyses. This means for determining differences in physical activity levels the significance was set at p <0.0167 (0.05/3) (SED, LPA and MVPA), for daily step count at *p* < 0.05 (Step count), and for the associations at *p* < 0.0167 (0.05/4) (Step count/SED/LPA/MVPA). Statistical analyses were conducted using SPSS (version 25; IBM SPSS Inc, Chigago, IL, USA).

## Results

### Participant Characteristics

Thirty-one adolescents with CP and 31 TD peers, together with 30 adults with CP and 30 TD peers were included in the research project. However, due to invalid wear time of the ActiGraph, 5 adolescents with CP, 6 TD adolescents and 8 adults with CP were excluded for further data analyses. The personal characteristics of the remaining participants were similar to the original cohorts as described in Salie et al. (15). In the current study 26 adolescents with CP (84% inclusion rate), 25 TD adolescents (81%), 22 adults with CP (73%) and 30 TD adults (100%) participated. In the adolescents with CP cohort, 8 (31%) were diagnosed with unilateral spastic CP, 14 (54%) with bilateral spastic CP, and 4 (15%) with dyskinesia. The adults with CP consisted of 2 (9%) who were diagnosed with unilateral spastic CP, 17 (77%) with bilateral spastic CP and 3 (14%) with dyskinesia. Table 1 provides an overview of the characteristics of all participants included in this study. Due to the purposeful matching of the CP and TD cohorts for age, sex and SES, there were no statistical differences between the two cohorts. Step count data from two adolescents with CP who were classified as GMFCS level V were identified as outliers and excluded from analysis, while PA data for these adolescents were retained.

**Table 1:** Overview of participant's characteristics expressed in median [interquartile ranges] or *n* (%).

	**Adolescents**		**Adults**	
**Parameters**	**CP (*n* = 26)**	**TD (*n* = 25)**	**CP (*n* = 22)**	**TD (*n* = 30)**
Age (years)	17.8 [16.8–18.5]	17.3 [16.9–18.0]	34.9 [30.9–36.3]	34.0 [29.3–35.6]
Sex				
Male	13 (50)	11 (44)	7 (32)	10 (33)
Female	13 (50)	14 (56)	15 (68)	20 (67)
GMFCS				
Level I	4 (15)	n/a	6 (27)	n/a
Level II	7 (27)		4 (18)	
Level III	6 (23)		5 (23)	
Level IV	4 (15)		4 (18)	
Level V	5 (19)		3 (14)	
MACS				
Level I	18 (69)	n/a	16 (73)	n/a
Level II	3 (12)		4 (18)	
Level III	2 (8)		1 (5)	
Level IV	2 (8)		1 (5)	
Level V	1 (4)		0 (0)	
CFCS				
Level I	19 (73)	n/a	18 (82)	n/a
Level II	6 (23)		3 (14)	
Level III	0 (0)		0 (0)	
Level IV	1 (4)		1 (5)	
Level V	0 (0)		0 (0)	
BMI	19.8 [18.1–25.5]	21.2 [19.0–25.0]	26.6 [23.1–36.8]	28.1 [22.9–32.9]
Underweight	8 (31)	4 (16)	1 (5)	1 (3)
Healthy	11 (42)	14 (56)	9 (41)	9 (30)
Overweight	4 (15)	6 (24)	4 (18)	11 (37)
Obese	3 (12)	1 (4)	8 (36)	9 (30)
SES	1.3 [1.0–1.8]	1.3 [0.9–1.6]	1.4 [1.0–1.7]	1.3 [1.0–1.7]
Low	3 (12)	6 (24)	4 (18)	3 (10)
Average	16 (62)	13 (52)	12 (55)	18 (60)
High	7 (27)	6 (24)	6 (27)	9 (30)

*CP, Cerebral Palsy; TD, Typically Developing; IQR, interquartile ranges; n/a, not applicable; GMFCS, Gross Motor Function Classification System; MACS, Manual Ability Classification System; CFCS, Communication Function Classification System; BMI, Body Mass Index; and SES, Socio Economic Status*.

### Wear Time

Of the 26 *adolescents* with CP, 19 (73%) recorded valid PA data for 7 days and 7 (27%) for 6 days, while of the 25 TD adolescents, 22 (88%) recorded PA data for 7 days, 1 (4%) for 6 days and 2 (8%) for 5 days. Calculated over the valid days, on average the recorded wear time (duration that the ActiGraph was worn for the day) had a median [IQR] of 14 h 30 m [13 h 51 m−15 h 18 m] for the adolescents with CP and 15 h 30 m [14 h 12 m−16 h 9 m] for the TD adolescents.

With regards to the *adults*, of the 22 adults with CP, 14 (64%) recorded PA data for 7 days, 6 (27%) for 6 days, and the remaining 2 (9%) recorded data for 5 and 4 days each. Of the 30 TD adults, 26 (86%) recorded PA data for 7 days, 2 (7%) for 6 days and 2 (7%) for 5 days. The actual valid median [IQR] time the adults wore the ActiGraphs per day (calculated as an average over all days) had a median [IQR] of 14 h 36 m [13 h 4 m−15 h 36 m] for adults with CP and 14 h 54 m [14h29m−16 h 21 m] for TD adults.

### Step Count

**Figure 1:**
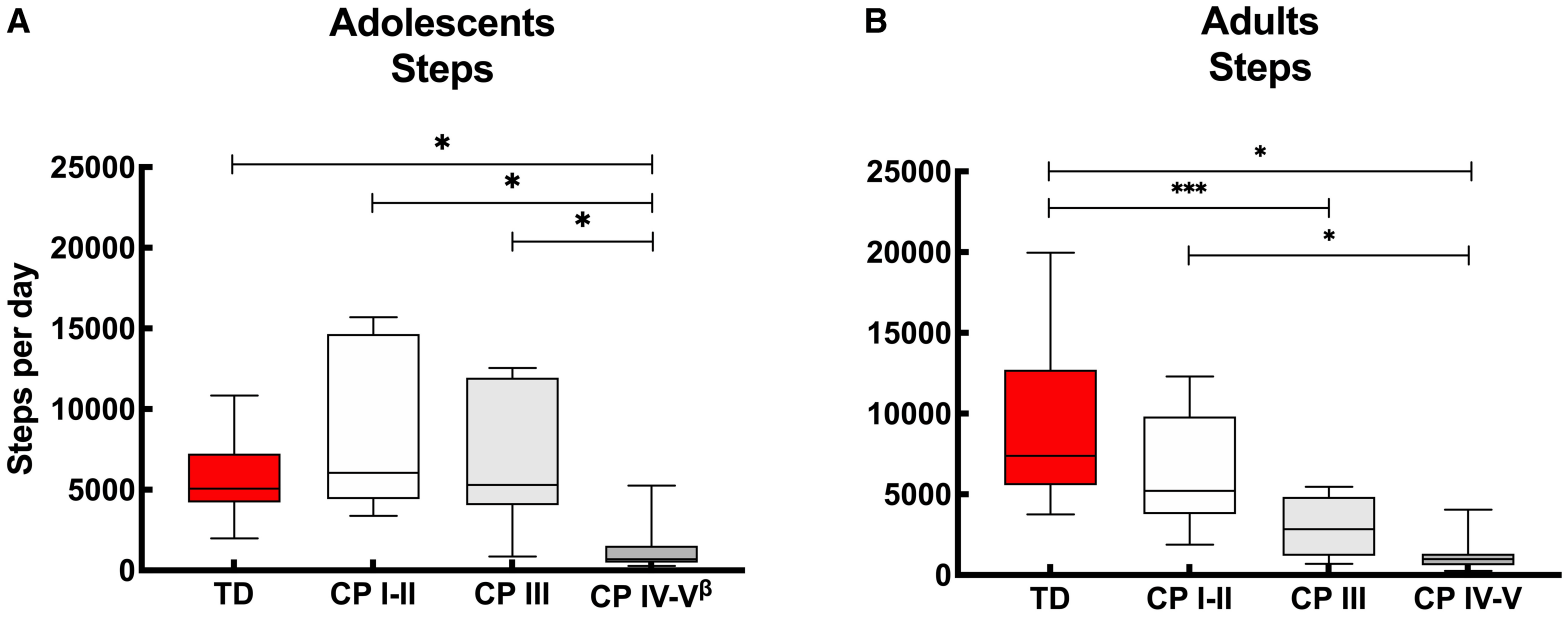
Box and whisker plots for daily step count of **(A)** adolescents and **(B)** adults with cerebral palsy (CP) and typically developing (TD) peers. CP groups include CP I-II, participants with CP classified in Gross Motor Functional Classification System (GMFCS) level I and II; CP III, participants with CP classified in GMFCS level III; CP IV-V, participants with CP classified in GMFCS level IV and V. ^ß^Data of 2 adolescents excluded: *n* = 24. ^*^Significant difference found for “whole week,” “weekdays” and “weekend days;” ^***^significant difference found for “whole week” and “weekdays” (*p* < 0.05).

With regards to the adolescents, on average over the “whole week,” no differences were reported in daily step count between the ambulant adolescents with CP (CP I-II and CP III) compared to the TD peers. On the other hand, the non-ambulant adolescents with CP (CP IV-V) showed lower step counts to the TD peers and the other CP groups. These results were the same for “weekdays” and “weekend days” only (Figure 1A, Appendix I).

With regards to the adult groups, the average step count calculated over the “whole week” was similar for the ambulant adults with CP (CP I-II) and TD peers, while it was lower for the other CP groups (CP III and CP IV-V) compared to the TD adolescents. The step count was also lower in the non-ambulant group (CP IV-V) compared to the independently ambulant adults with CP (CP I-II). Overall, the results were similar calculated over the “whole week,” “weekdays” and “weekend days,” except with the step count between the adults with CP who walked with assistive devices (CP III) and TD peers which was not significant different during the “weekend days” (Figure 1B, Appendix I).

### Levels of Physical Activity

The results of PA are recorded in appendices, including data from the adolescent's groups using Freedson (Appendix IIa) and Evenson (Appendix IIb) cut-point, as well as the adult's groups with Freedson (Appendix IIIa) and Evenson cut-points (Appendix IIIb). For clarity, the results described below are based on the data calculated with the Freedson cut-points. Despite the fact that Freedson cut-points (25) were only validated in adult population, the data is very similar to outcomes where Evenson cut-points (26) were utilized.

**Figure 2:**
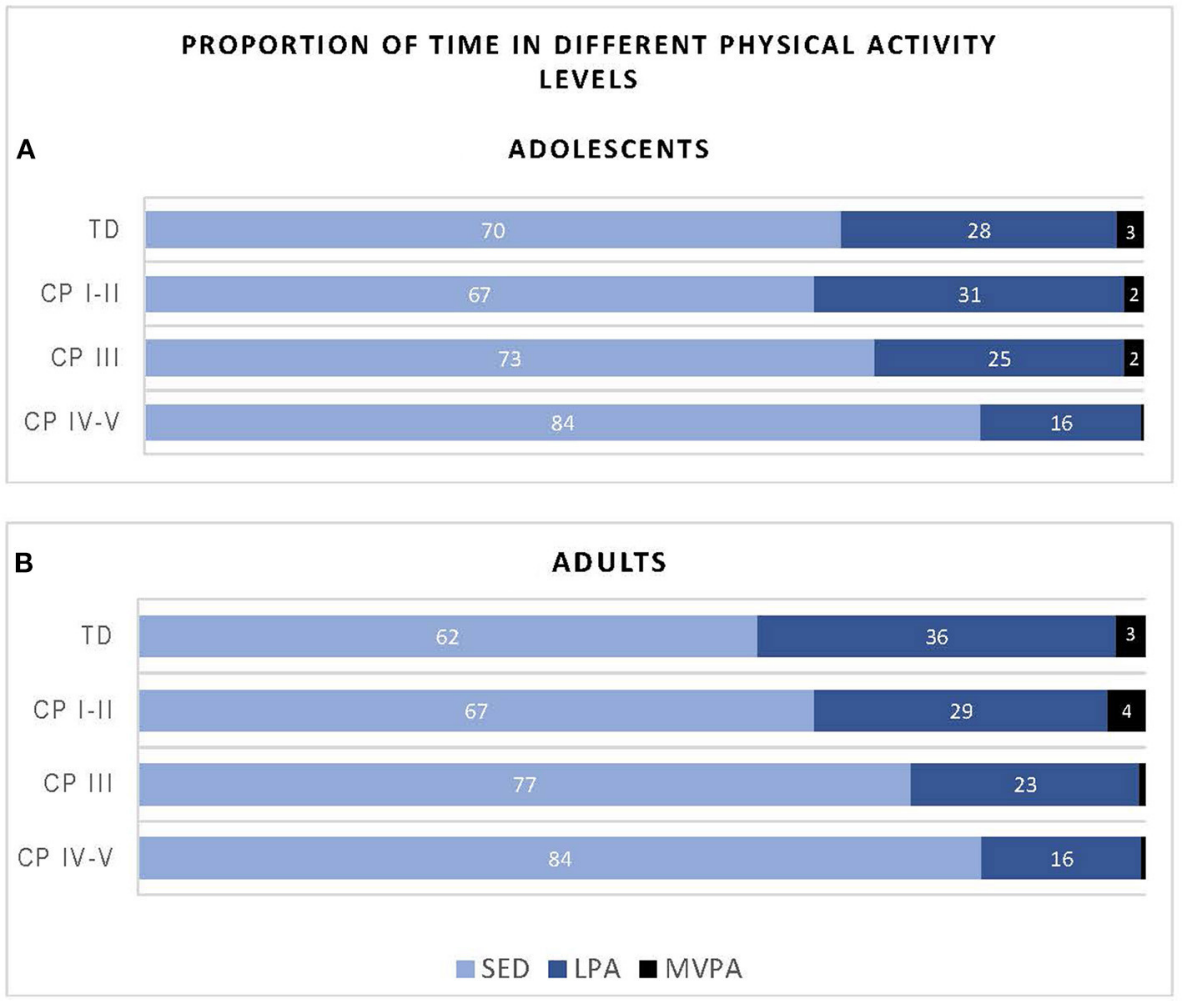
Proportion of time adolescents **(A)** and adults **(B)** with cerebral palsy (CP) and typically developing (TD) peers spend in sedentary (SED), low physical activity (LPA) and moderate to vigorous physical activity (MVPA) based on data recorded over the “whole week” using Freedson cut-points. CP groups includes CP I-II, participants with CP classified in Gross Motor Functional Classification System (GMFCS) level I and II; CP III, participants with CP classified in GMFCS level III; CP IV-V, participants with CP classified in GMFCS level IV and V.

Figure 2 shows the proportion of time the participants of the different groups spent in SED, LPA and MVPA. Overall, adolescents with CP (classified in GMFCS level I-V) spend on average 74% of their day in SED, 24% in LVPA and 1% in MVPA, while their TD peers spend 70% of the time in SED, 28% in LPA and 3% in MVPA. With regards to the adults with CP, all adults with CP together (GMFCS level CP I-V) spend on average 75% of their time in SED, 23% in LPA and 2% in MVPA, while the TD peers spend 62% of the time in SED, 36% in LPA and 3% in MVPA.

**Figure 3:**
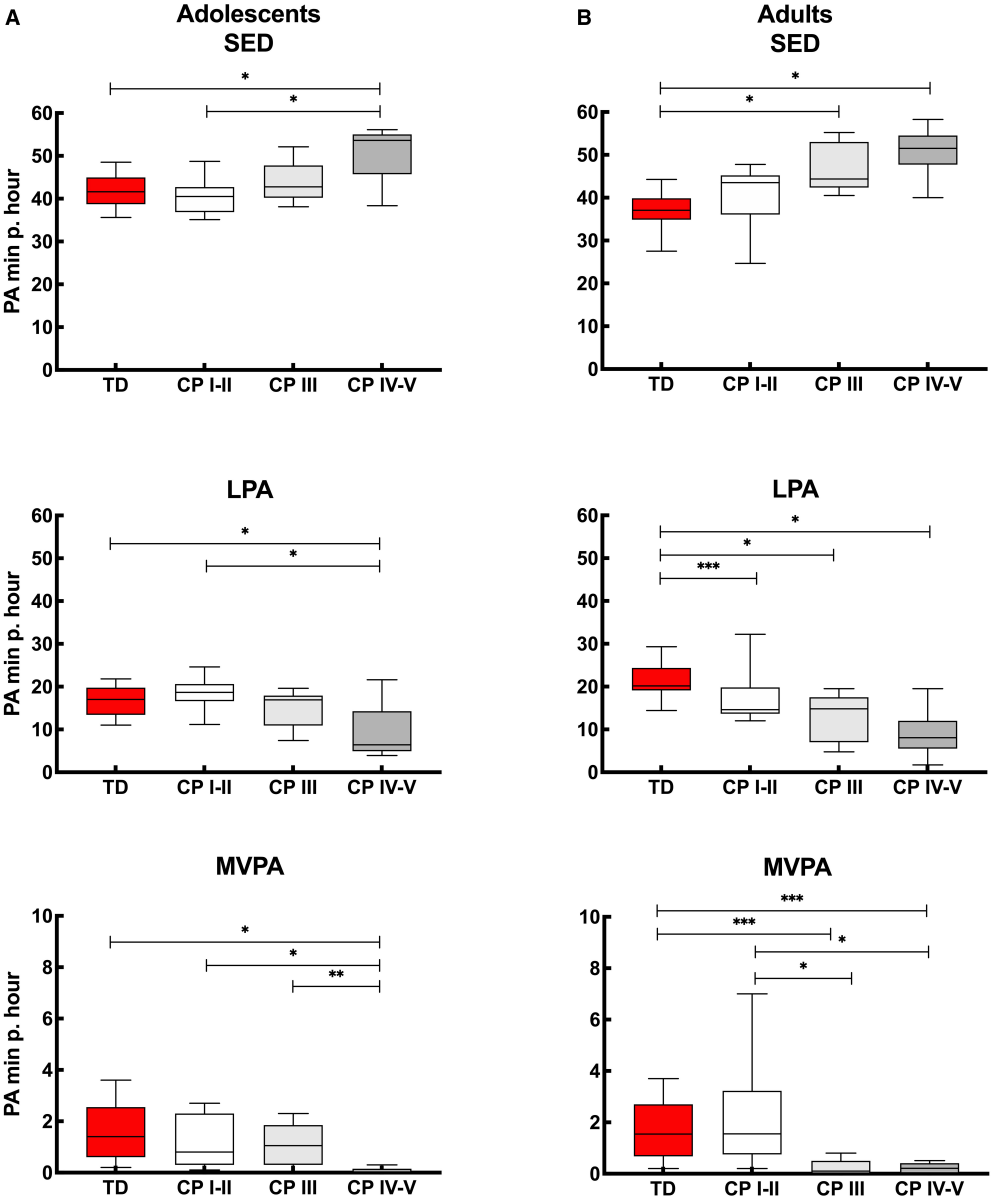
Box and whisker plots for time spent in sedentary (SED), low physical activity (LPA) and moderate to vigorous physical activity (MVPA) using Freedson cut-points of **(A)** adolescents and **(B)** adults with cerebral palsy (CP) and typically developing peers (TD). CP groups include CP I-II, participants with CP classified in Gross Motor Functional Classification System (GMFCS) level I and II; CP III, participants with CP classified in GMFCS level III; CP IV-V, participants with CP classified in GMFCS level IV and V. ^*^Significant difference found for “whole week,” “weekdays” and “weekend days;” ^**^significant difference found for “whole week” and “weekend days;” ^***^significant difference found for “whole week” and “weekdays” (*p* < 0.0167).

Figure 3A provides insight into the actual time (minutes per hour) the adolescents spend in SED, LPA and MVPA. Calculated over the “whole week,” the PA levels of adolescents with CP who are ambulant (CP I-II and CP III) were similar to the TD peers. Adolescents with CP who were non-ambulant (CP IV-V) spent more time SED and less time in LPA and MVPA compared to adolescents with CP who were independently ambulant (CP I-II) and TD adolescents. In addition, adolescents with CP who were non-ambulant (CP IV-V) spent less time in MVPA than adolescents with CP who were using assistive devices (CP III) (Figure 3A, Appendix IIa). Overall, similar results were observed when interpreting the data for the “whole week,” “weekdays” and “weekend days.” The only difference found was that the adolescents with CP who used assistive devices (CP III) spent more time in MVPA than non-ambulant adolescents with CP (CP IV-V) during the “whole week” and “weekend,” but this was not the case for “weekdays” (Figure 3A, Appendix IIa).

The results of the *adults* with CP and TD peers are shown in Figure 3B. When considering the “whole week,” then no differences were found in PA levels between independently ambulant adults with CP (CP I-II) and TD peers for SED and MVPA, while the level of PA (based on SED, LPA and MVPA) was lower in the other CP groups (CP III and CP IV-V) compared to TD peers. In addition, MVPA was higher in the CP I-II group compared to CP III and CP IV-V groups. Results of ‘whole week’ were quite similar to the outcomes calculated over “weekdays” and “weekend days.” The exception being LPA levels, which were not lower in CP I-II compared to TD during the “weekend days” and MVPA was not lower in CP III and CP IV-V compared to TD adults when the results of the weekend days only were considered (Figure 3B, Appendix IIIa).

### Associations

As shown in Table 2, adolescents and adults with CP both showed an association between daily step count and GMFCS levels, which indicates that adolescents and adults who were less independently mobile showed a lower daily step count. In line with this, higher GMFCS levels in adolescents and adults with CP was associated with greater time spent SED and a lower time spent in MVPA. No associations were found between daily step count or PA with sex, BMI and SES.

**Table 2:** Overview of the associations between step count and level of physical activity (minutes per hour)^#^ and contextual factors for adolescents (*n* = 26) and adults (*n* = 22) with cerebral palsy.

**Parameters**		**Contextual factors**			
		**Sex**	**GMFCS**	**BMI**	**SES**
**Adolescents**					
Step count^ß^	rho	−0.260	-0.614	−0.202	0.179
	*p*	0.220	0.001^*^	0.344	0.402
SED	rho	0.241	0.574	0.145	0.006
	*p*	0.235	0.002^*^	0.479	0.978
LPA	rho	−0.159	-0.536	−0.197	0.004
	*p*	0.438	0.005^*^	0.334	0.984
MVPA	rho	−0.477	-0.636	0.204	−0.109
	*p*	0.014	<0.001^*^	0.319	0.598
**Adults**					
Step count	rho	0.315	-0.765	−0.440	0.373
	*p*	0.153	<0.001^*^	0.041	0.087
SED	rho	−0.331	0.572	0.375	−0.264
	*p*	0.133	0.005^*^	0.085	0.235
LPA	rho	0.331	-0.528	−0.354	0.166
	*p*	0.132	0.012^*^	0.106	0.460
MPA	rho	0.355	-0.746	−0.311	0.522
	*p*	0.105	<0.001^*^	0.159	0.013

# *Average physical activity measured over the “whole week” using Freedson cut-points. GMFCS, Gross Motor Classification System; BMI, Body Mass Index; SES, socio-economic status; SED, sedentary; LPA, Low physical activity; MVPA, Moderate to vigorous physical activity*.

ß *Data of 2 adolescents excluded: n = 24*.

* *Significance set at p < 0.0125*.

## Discussion

Despite what has been previously reported in well-resourced environments (HIC), as well as the hypothesis for the current study conducted in LMIC, not all adolescents and adults with CP were less active than their peers. This study demonstrated that PA in adolescents and adults with CP classified in GMFCS level I-II are no different to their TD peers when considering the whole week. Outcomes were similar for adolescents with CP who walked with assistive devices (GMFCS level III), however, *adults* with CP classified in GMFCS level III showed a lower daily step count and less PA than TD adults. Understandably, both adolescents and adults with CP who are non-ambulant (GMFCS level IV-V), showed lower daily step counts as well as lower PA levels compared to the TD cohorts. These results confirm the inverse relationship between participant's GMFCS levels and PA, indicating that adolescents and adults with CP who are less independently mobile show reduced physical activity.

### Valid Data

In the current study, accelerometry data was captured with ActiGraph GT3X (worn around the waist). The ActiGraph has been considered the gold standard for PA research in individuals with CP as well as the TD population (27–31). The protocol we used was based on a systematic review (24) and other similar studies (27, 28, 34), which led to a relatively good succession rate with 73–100% of the CP and TD cohorts having valid data and included in the study. This data was based on 7 days of the week in 79% of all cases (6 days: 16; 5 days: 5; and 4 days: 1%), which corresponded with a real presentation of PA for a week.

### Step Count

No differences were found between the daily step count for ambulant adolescents with CP (classified in GMFCS levels I-II and III) and TD peers (Figure 1A, Appendix I). This contrasts with previous findings from studies conducted in HIC (37, 38) reporting adolescents with CP to have a lower daily step count than TD adolescents. A possible reason for the lack of consistency of findings across these studies may include the relatively low daily step count of the TD peers in the current study (median: 5,075 steps), compared to the other studies with average step counts of 10,100 (38) and 6,812 (37). An alternative explanation is that the ambulant adolescents with CP in the current study had a relatively high daily step count. The adolescents with CP who used an assistive device (GMFCS level III), in particular, had a relatively high median daily step count of 5,310 steps, while the other studies reported 2,574 (9) and 2,606 (38) steps. These differences could be due to the use of other accelerometers ActivPal 3 (9, 38) and Orthocare SAM (37) or may be explained by social and physical environment factors (12). The fact that the adolescents with CP who reside in a LMIC have a relative high step count, may be due to challenges they face with accessing transport, whether private or public, and may have to resort to walking in order to get to their destination. Financial challenges may also play a role in choosing to walk instead of taking a bus or taxi for transportation.

In line with the adolescents, considering the whole week, independent ambulant adults with CP (GMFCS level I-II) had a similar step count to TD adults, though adults who walked with assistive devices (GMFCS level III) had a lower step count than their TD peers. Step count for this group was different for whole week and weekdays, with no differences between adults with GMFCS level III and their TD peers in daily step count during the weekend. This suggests that these adults are capable of a typical daily step count but were not achieving this during the week. This is in contrast to the non-ambulant adults with CP (GMFCS level IV-V), who understandably did not achieve a daily step count similar to TD peers (Figure 1B, Appendix I).

### Physical Activity

In line with the daily step count results, the current study showed that PA levels for *adolescents* with CP who are ambulant as well as those who used assistive devices, residing in a LMIC such as South Arica, are similar to TD peers (Figure 2A, Appendix IIa). These findings differ from previous studies conducted among adolescents with CP who reside in HICs, who reported more sedentary behavior in adolescents with CP compared to TD adolescents (10, 37, 38).

A more obvious finding of the current study was the difference in time spent in SED, LPA and MVPA in non-ambulant adolescents with CP compared to ambulant participants. More specifically, the current study showed that adolescents classified in GMFCS level IV-V reported less physical activity than TD adolescents and adolescents with CP classified in GMFCS level I-II and level III. The difference in MVPA between adolescents classified in GMFCS level III compared to GMFCS level IV-V was not found during the weekdays only, explained by a relatively low MVPA in this group. This highlights the fact that those adolescents with CP who used assistive devices in the current study are capable of being more active (evident on the weekends). A perceived barrier for reduced PA during the weekdays could be ignorance, due to lack of knowledge and active-living skills (11). Adolescents should be encouraged to be active to prevent long-term health issues (8, 11).

When comparing the PA outcomes of the adolescents with CP of current study with the work from Gorter and colleagues (31), who used a similar protocol (also including the Actigraph), but conducted in Canada, similar findings were reported. Gorter and colleagues (31) also showed lower LPA and MVPA for non-ambulant adolescents (GMFCS level IV) compared to independently ambulant adolescents (GMFCS level I and level II). However, it seems that the adolescents in the latter study (27) spent more time in MVPA (mean time per GMFCS level I/II/III/IV: 4.5/2.2/1.5/0.5 minutes per hour), compared to the current study (median time per GMFCS level I-II/III/IV-V: 0.8/1.1/0.0 minutes per hour). This shows that overall, the CP cohort living in urban South Africa was less active than the Canadian cohort.

Similar to the findings reported in the adolescent group, the independently ambulant *adults* with CP (GMFCS level I-II) showed similar PA levels (SED and MVPA) to their TD peers (Figure 3, Appendix IIIa). This is in contrast to adults with CP classified in GMFCS level III and level IV-V, who were less active than the TD adults. Nieuwenhuijsen and colleagues (39) similarly reported that adults with CP living in the Netherlands were relatively inactive compared to their peers, especially for those classified in GMFCS level III and IV (GMFCS level V was not included in the study), in agreement with our findings.

The difference in time spent in MVPA for adults with CP who used assistive devices and the non-ambulant adults with CP compared to TD peers was not seen on the weekends. This was because a relatively short time was spent in MVPA by the TD adults in the current study during the weekend. This highlights the fact that not only should adults with CP be motivated to spend more time in MVPA, but also the TD population. Therefore, TD adults may need to be encouraged to be more active during the weekend as they are also at risk for non-communicable disease (NCD) such as stroke, heart disease or diabetes (40).

When comparing the level of PA within the CP groups, then the current study reported no differences in SED and LPA between ambulant adults with CP (GMFCS level I-III) with non-ambulant adults (GMFCS level IV-V). On the other hand, differences between CP groups were found for MVPA as also previously reported in the literature. Ryan and colleagues (41) reporting on a cohort from Ireland and using a RT3 accelerometer, found differences between ambulant adults with CP classified in GMFCS level I compared to GMFCS levels II and level III. In addition, Claridge and colleagues (27) in a study conducted in Canada which also used the ActiGraph in ambulant and non-ambulant adults with CP, reported differences between ambulant adults with CP (GMFCS levels I, II and III) to non-ambulant adults (GMFCS level IV and level V).

The latter (27) was in contrast to the current study, with no differences found between adults who walk with an assistive device (GMFCS level III) and non-ambulant adults (GMFCS level IV-V). This finding could possibly be explained by the fact that relatively more time was spent in MVPA by adults classified in GMFCS level III in the Canadian study (MVPA GMFCS level I/II/III/IV/V: 3.8 / 1.6 / 0.7 / 0.1 / 0.1 minutes per hour) compared to the current study (MVPA per GMFCS level I-II/III/IV-V: 1.6 / 0.1 / 0.2 minutes per hour).

The differences between the outcomes of the adolescents and adults with CP, particularly those who used assistive devices, compared to their peers may suggest that as individuals with CP grow into adulthood, they tend to be less physically active. This is in line with the decline in walking ability which was shown especially for those who are less independent in gait (42) and started as early as mid-30's in individuals with CP (43). Intrinsic (e.g., pain, fatigue) and extrinsic factors (e.g., environment) could be the cause of walking restrictions (44) and should try to be prevented. Walltersson and colleagues (6) suggest that when adolescents with CP are physically active in their youth, that this increases the likelihood of having higher PA levels in adulthood. Therefore, participation in regular exercise programs from an early age must be educated and encouraged in order to promote increased PA in adulthood so that the challenges and secondary complications of aging with a disability could be minimized. PA programs with theoretical and practical knowledge about the benefits of PA will help developing life-skills, which is of value for the whole lifespan (11). Though the barriers during the transition phase and thereafter are not only experienced by the individual with CP, there are also challenges for the health care system and clinicians, which needs to be addressed (45).

Regular PA not only benefits physical fitness but also cardiometabolic health (blood pressure, diabetes), bone health, mental health (anxiety and depression), maintain healthy body weight as well as cognitive function (40). Therefore, the World Health Organisation (WHO) 2020 guidelines (46), recommend adolescents, whether or not disabled, to engage in a minimum of 60 min per day on average of moderate- to- vigorous intensity, aerobic physical activity. Furthermore, the guidelines for adults, with or without a disability, are similar and include a recommendation to spend a minimum of 150 to 300 min of moderate intensity or 75 to 150 min vigorous intensity aerobic physical activity per week. Data from current study provides PA results based on minutes per hour and not per day, and therefore does not allow one to determine whether the CP and TD cohorts fulfill these criteria. However, with all cohorts spending only 1–3% of an h in MVPA (adolescents: CP 1 and TD 3%, adults: CP 2 and TD 3%) (Figure 1), it is clear that on average the adolescents and adults with CP and TD peers living in urban South Africa do not meet the WHO PA criteria.

### Associations

As expected, the daily step count and all levels of PA were associated with GMFCS levels for both adolescents and adult with CP, indicating that participants with CP who were less independently mobile (higher GMFCS level) took fewer steps and spent less time being physically active (Table 2). This is corroborated by previous studies conducted with adolescents (9, 31) and adults (39) with CP. In line with the findings of Nieuwenhuijsen and colleagues (39), no associations were found for sex, BMI and SES, between PA levels and personal or environmental factors, with the exception of GMFCS levels.

### Limitations

Several limitations should be considered when interpreting the results of this study. Although the small sample size for the different adolescent and adult CP groups (based on GMFCS levels) was relatively small, the groups were well distributed (Table 1) and personal characteristics were similar to the original cohorts (15). Another point of discussion is the use of cut-points to determine PA levels. The results presented are based on the data created with outcomes using Freedson cut-points. As mentioned, Freedson cut-points are developed for adults (25), while Evenson cut-points for children (26). Despite the differences in cut-points, especially for time spent in MVPA, we found minimal differences in the outcomes when interpretating the data with Freedson (Appendices IIa, IIIa) and Evenson (Appendices IIb, IIIb) cut-points. In addition, Freedson and Evenson cut-points have been validated in the general population and this may have underrepresented activity levels in those with lower gross motor function (e.g., GMFCS levels III to V). Likewise, because the ActiGraph was worn around the waist, activity in the upper limbs may not have been accounted for when determining PA, which may have affected PA levels of individuals with CP who walked with assistive devices (GMFCS level III) or those who were non-ambulant (GMFCS level IV and level V) but have been self-propelling their wheelchairs. No logbook or other questionnaire was completed, which could have been used to check the reliability of the data. Another limiting factor is that this is not a longitudinal study, where adolescents with CP were followed into adulthood, but rather based on different cohorts during adolescence and adulthood. Future follow-up studies will provide more insight into a possible trajectory although we can anticipate and plan for future challenges faced during the transition phase. In addition, the current study has been conducted in urban South Africa and one can hypothesize that the outcomes might differ for adolescents and adults living in rural communities with a different distribution of resources. Therefore, the outcomes cannot be generalized for all individuals with CP living in LMICs who may have different cultural norms and regulations or policies in place.

## Conclusion

The current study showed that daily step count and PA levels of ambulant adolescents with CP, classified as GMFCS levels I to III, were similar to TD peers when considering the whole week. In contrast, ambulant *adults* with CP who used assistive devices (GMFCS level III) were less physically active than TD peers. Adolescents and adults with CP who were non-ambulant (GMFCS level IV-V) reported less daily step count and levels of PA compared to their peers. In addition, this study confirmed an inverse relationship between participant's GMFCS levels and PA, while the contextual factors of sex, BMI and SES were not related.

Since the level of PA in adolescence might be a predictor for PA in adulthood (6), providing support and intervention to this group during transition would be critical to maintain function (not only in the weekends as seen in the current study, but also during the week) in order to promote physical health, social and emotional well-being and independence. Even though physical challenges may hamper participation in PA, individuals with CP should be encouraged, assisted and supported to meet the PA recommendations by the WHO (40, 47). These should be developmentally appropriate, enjoyable, and involve a variety of activities (11, 48). While this is a global aspiration, it seems that adolescents and adults with CP living in a LMIC such as South Africa, spend relatively less time in MVPA than peers living in HIC. It is also important to note that the overall PA level of the TD population in the current study was lower than expected, especially during the weekend days, and this group may also benefit from encouragement to engage in regular PA. This indicates that activity promotion programs should not only be focused on the individuals with CP (as rehabilitation), but rather be part of a general healthy aging program for the whole community.

## Data Availability Statement

The original contributions presented in the study are included in the article/Supplementary Material, further inquiries can be directed to the corresponding author/s.

## Ethics Statement

The studies involving human participants were reviewed and approved by Human Research Ethics Committee, Faculty of Health Sciences, University of Cape Town, South Africa. Written informed consent to participate in this study was provided by the participants or participants' legal guardian/next of kin.

## Author Contributions

NL and RS conceived and designed the analysis. RS and ME collected the data. RS, NL, and ME performed the analysis and drafted the manuscript. RS, NL, ME, KD, and AF provided critical feedback and contributed to the final manuscript. All authors contributed to the article and approved the submitted version.

## Funding

This work was supported by the National Research Foundation, Pretoria, South Africa as well as the Neuroscience Institute, University of Cape Town, South Africa.

## Conflict of Interest

The authors declare that the research was conducted in the absence of any commercial or financial relationships that could be construed as a potential conflict of interest.

## Publisher's Note

All claims expressed in this article are solely those of the authors and do not necessarily represent those of their affiliated organizations, or those of the publisher, the editors and the reviewers. Any product that may be evaluated in this article, or claim that may be made by its manufacturer, is not guaranteed or endorsed by the publisher.
