# Supplementary material for: Physical Activity Levels of Adolescents and Adults With Cerebral Palsy in Urban South Africa
Source: Front Neurol. 2021 Oct 28;12:747361. doi: 10.3389/fneur.2021.747361 (PMC8581637; doi:10.3389/fneur.2021.747361)
Supplement: Supplementary file 1 [file Data_Sheet_1.docx]

| Appendix I: Overview of step count of **adolescents** and **adults** with cerebral palsy (CP) and typically developing (TD) peers and an indication of group differences. | | | | | | | | | | | | | |
| --- | --- | --- | --- | --- | --- | --- | --- | --- | --- | --- | --- | --- | --- |
| Parameters | TD | CP I-II | CP III | CP IV-V | *Overall Group effect* | TD vs  CP I-II | TD vs  CP III | TD vs  CP IV-V | | CP I-II vs  CP III | CP I-II vs  CP IV-V | CP III vs  CP IV-V |  |
| (min/hour) | Median  [IQR] | Median  [IQR] | Median  [IQR] | Median  [IQR] | *p* | *p* | *p* | | *p* | *p* | *p* | *p* |  |
| **Adolescents** |  |  |  |  |  |  |  | |  |  |  |  |  |
| Whole week | 5075  [4209 - 7230] | 6053  [4426 – 14648] | 5310  [4059 – 11951] | 703  [497 –1521] | ***0.002**** | 0.155 | 0.579 | ***0.002**** | | 0.606 | ***<0.001**** | ***0.005**** |  |
| Weekdays | 5365  [4268 - 7981] | 6443  [4310 – 16672] | 5546  [4206 – 13165] | 776  [619 – 1610] | ***0.001**** | 0.152 | 0.627 | ***0.002**** | | 0.557 | ***<0.001**** | ***0.005**** |  |
| Weekend days | 4505  [2327-6078] | 5077  [4783 – 8030] | 4826  [3533 – 8918] | 536  [234 – 1298] | ***0.004**** | 0.167 | 0.562 | | ***0.005**** | 0.641 | ***<0.001**** | ***0.008**** |  |
| **Adults** |  |  |  |  |  |  |  | |  |  |  |  |  |
| Whole week | 7382  [5565 – 12712] | 5220  [3776 – 9814] | 2703  [968 – 4221] | 983  [522 – 1350] | ***<0.001**** | 0.092 | ***0.002**** | | ***<0.001**** | 0.106 | ***0.010**** | 0.507 |  |
| Weekdays | 8636  [6292 – 14622] | 5165  [3043 – 10824] | 3042  [803 – 4397] | 1038  [429 - 1459] | ***<0.001**** | 0.066 | ***0.001**** | | ***<0.001**** | 0.089 | ***0.013**** | 0.609 |  |
| Weekend days | 5323  [3490 – 8497] | 5618  [2261 – 7900] | 1640  [1258 – 3889] | 707  [449 – 1076] | ***<0.001**** | 0.842 | 0.020 | | ***<0.001**** | 0.055 | ***0.001**** | 0.329 |  |
| *Abbreviations*: TD, Typically Developing; CP, Cerebral Palsy; CP I-II, participants with CP classified in Gross Motor Functional Classification System (GMFCS) level I and II; CP III, participants with CP classified in GMFCS level III; CP IV-V, participants with CP classified in GMFCS level IV and V; and IQR, Interquartile ranges. *Significant difference with *p*<0.05. | | | | | | | | | | | | | |

| Appendix IIa: Overview of time (in minutes per hour) **adolescents** with cerebral palsy (CP) and typically developing (TD) peers spent in sedentary, low and middle to vigorous physical activity levels using cut-offs defined by **Freedson** and an indication of group differences. | | | | | | | | | | | |
| --- | --- | --- | --- | --- | --- | --- | --- | --- | --- | --- | --- |
| Parameters | TD  (n=25) | CP I-II  (n=11) | CP III  (n=6) | CP IV-V  (n=7) | *Overall Group effect* | TD vs  CP I-II | TD vs  CP III | TD vs  CP IV-V | CP I-II vs  CP III | CP I-II vs  CP IV-V | CP III vs  CP IV-V |
| (min/hour) | Median  [IQR] | Median  [IQR] | Median  [IQR] | Median  [IQR] | *p* | *p* | *p* | *p* | *p* | *p* | *p* |
| **Whole week** | | | | | | | | | | | |
| SED | 41.6  [38.7 - 45.0] | 40.5  [36.9 – 42.7] | 42.8  [40.2 – 47.8] | 53.6  [45.7 – 55.0] | ***0.002**** | 0.316 | 0.479 | ***0.001**** | 0.177 | ***<0.001**** | 0.760 |
| LPA | 17.0  [13.5 - 19.8] | 17.8  [15.0 – 19.4] | 16.0  [10.7 - 17.8] | 6.2  [5.0 – 14.1] | ***0.008**** | 0.412 | 0.414 | ***0.003**** | 0.188 | ***0.001**** | 0.146 |
| MVPA | 1.4  [0.6 - 2.5] | 0.8  [0.3 – 2.3] | 1.1  [0.4 – 1.9] | 0.0  [0.0 – 0.2] | ***<0.001**** | 0.259 | 0.309 | ***<0.001**** | 0.914 | ***0.002**** | ***0.012**** |
| **Weekdays** | | | | | | | | | | | |
| SED | 43.5  [39.0 - 46.6] | 41.4  [37.1 – 45.7] | 44.6  [41.1 – 48.6] | 53.4  [46.4 – 56.6] | ***0.004**** | 0.398 | 0.406 | ***0.002**** | 0.178 | ***0.001**** | 0.113 |
| LPA | 16.4  [13.4 - 18.9] | 18.6  [17.0 – 22.1] | 16.7  [11.7 – 18.2] | 6.4  [5.5 – 14.0] | ***0.004**** | 0.110 | 0.629 | ***0.007**** | 0.116 | ***<0.001**** | 0.119 |
| MVPA | 1.4  [0.7 – 2.6] | 1.0  [0.4 – 2.5] | 1.0  [0.1 – 2.0] | 0.0  [0.0 – 0.2] | ***<0.001**** | 0.361 | 0.205 | ***<0.001*** | 0.629 | ***0.002**** | 0.031 |
| **Weekend days** | | | | | | | | | | | |
| SED | 40.9  [36.2 – 44.1] | 40.1  [37.3 – 44.5] | 43.6  [39.5 – 49.3] | 52.2  [45.1 – 55.7] | ***0.010**** | 0.953 | 0.282 | ***0.001**** | 0.357 | ***0.007**** | 0.153 |
| LPA | 17.7  [14.8 – 20.4] | 19.3  [15.3 – 21.8] | 16.4  [8.9 – 19.1] | 7.8  [4.3 – 14.9] | ***0.015**** | 0.750 | 0.330 | ***0.003**** | 0.271 | ***0.005**** | 0.180 |
| MVPA | 0.7  [0.3 – 1.7] | 0.3  [0.2 – 1.2] | 1.3  [0.1 – 1.9] | 0.0  [0.0 – 0.1] | ***<0.001**** | 0.436 | 0.837 | ***<0.001**** | 0.711 | ***0.003**** | ***0.004**** |
| *Abbreviations*: TD, Typically Developing; CP, Cerebral Palsy; CP I-II, participants with CP classified in Gross Motor Functional Classification System (GMFCS) level I and II; CP III, participants with CP classified in GMFCS level III; CP IV-V, participants with CP classified in GMFCS level IV and V; IQR, Interquartile ranges; SED, sedentary; LPA, Low physical activity; MVPA, Moderate to vigorous physical activity. *Significant difference with *p*< 0.0167. | | | | | | | | | | | |

| Appendix IIb: Overview of time (in minutes per hour) **adolescents** with cerebral palsy (CP) and typically developing (TD) peers spent in sedentary, low and middle to vigorous physical activity levels using cut-offs defined by **Evenson** and an indication of group differences. | | | | | | | | | | | | |
| --- | --- | --- | --- | --- | --- | --- | --- | --- | --- | --- | --- | --- |
| Parameters | TD  (n=25) | CP I-II  (n=11) | CP III  (n=6) | CP IV-V  (n=7) | *Overall Group effect* | TD vs  CP I-II | TD vs  CP III | TD vs  CP IV-V | CP I-II vs  CP III | CP I-II vs  CP IV-V | CP III vs  CP IV-V |  |
| (min/hour) | Median  [IQR] | Median  [IQR] | Median  [IQR] | Median  [IQR] | *p* | *p* | *p* | *p* | *p* | *p* | *p* |  |
| **Whole week** | | | | | | | | | | | | |
| SED | 41.7  [38.8 - 45.0] | 40.5  [37.0 – 42.8] | 43.0  [40.3 – 47.8] | 53.6  [45.7 – 55.1] | ***0.002**** | 0.316 | 0.479 | ***0.001**** | 0.177 | ***<0.001**** | 0.076 |  |
| LPA | 17.3  [14.0 - 20.4] | 19.1  [16.8 – 21.3] | 16.9  [11.2 – 18.6] | 6.4  [4.9 – 14.3] | ***0.005**** | 0.412 | 0.414 | ***0.003**** | 0.188 | ***0.001**** | 0.146 |  |
| MVPA | 0.9  [0.4 - 1.8] | 0.4  [0.2 – 1.4] | 0.7  [0.2 – 1.2] | 0.0  [0.0 – 0.1] | ***<0.001**** | 0.218 | 0.216 | ***<0.001**** | 0.817 | ***0.004**** | 0.027 |  |
| **Weekdays** | | | | | | | | | | | | |
| SED | 42.3  [38.6 – 45.1] | 39.6  [35.0 – 41.9] | 42.6  [40.7 – 47.3] | 53.6  [46.0 – 54.6] | ***0.002**** | 0.209 | 0.631 | ***0.002**** | 0.185 | ***<0.001**** | 0.054 |  |
| LPA | 16.7  [13.7 – 19.5] | 19.1  [17.3 – 22.9] | 16.9  [12.1 – 18.5] | 6.4  [5.4 – 13.9] | ***0.003**** | 0.136 | 0.677 | ***0.005**** | 0.151 | ***<0.001**** | 0.085 |  |
| MVPA | 1.1  [0.5 – 2.0] | 0.5  [0.1 – 1.5] | 0.7  [0.1 – 1.3] | 0.0  [0.0 – 0.1] | ***<0.001**** | 0.218 | 0.216 | ***<0.001**** | 0.817 | ***0.004**** | 0.027 |  |
| **Weekend days** | | | | | | | | | | | | |
| SED | 41.0  [36.2 - 44.2] | 40.4  37.4 – 44.5] | 43.9  [39.6 – 49.3] | 52.2  [45.1 – 55.7] | ***0.010**** | 0.934 | 0.285 | ***0.001**** | 0.369 | ***0.007**** | 0.148 |  |
| LPA | 17.8  [15.2 - 21.0] | 19.3  [15.3 – 22.3] | 16.2  [9.0 – 19.7] | 7.8  [4.3 – 14.9] | ***0.013**** | 0.832 | 0.288 | ***0.003**** | 0.270 | ***0.005**** | 0.190 |  |
| MVPA | 0.4  [0.2 – 1.2] | 0.2  [0.1 – 0.8] | 0.9  [0.0 – 1.3] | 0.0  [0.0 – 0.0] | ***0.001**** | 0.368 | 0.708 | ***<0.001**** | 0.760 | ***0.005**** | ***0.008**** |  |
| *Abbreviations*: TD, Typically Developing; CP, Cerebral Palsy; CP I-II, participants with CP classified in Gross Motor Functional Classification System (GMFCS) level I and II; CP III, participants with CP classified in GMFCS level III; CP IV-V, participants with CP classified in GMFCS level IV and V; IQR, Interquartile ranges; SED, sedentary; LPA, Low physical activity; MVPA, Moderate to vigorous physical activity. *Significant difference with *p*< 0.0167. | | | | | | | | | | | | |

| Appendix IIIa: Overview of time (in minutes per hour) **adults** with cerebral palsy (CP) and typically developing (TD) peers spent in sedentary, low and middle to vigorous physical activity levels using cut-offs defined by **Freedson** and an indication of group differences. | | | | | | | | | | | |
| --- | --- | --- | --- | --- | --- | --- | --- | --- | --- | --- | --- |
| Parameters | TD  (n=30) | CP I-II  (n=10) | CP III  (n=5) | CP IV-V  (n=7) | *Overall Group effect* | TD vs  CP I-II | TD vs  CP III | TD vs  CP IV-V | CP I-II vs  CP III | CP I-II vs  CP IV-V | CP III vs  CP IV-V |
| (min/hour) | Median  [IQR] | Median  [IQR] | Median  [IQR] | Median  [IQR] | *p* | *p* | *p* | *p* | *p* | *p* | *p* |
| **Whole week** | | | | | | | | | | | |
| SED | 37.1  (34.9 – 39.9) | 43.5  (36.1 – 45.2) | 44.4  (42.4 – 53.0) | 51.5  (47.7 – 54.5) | ***<0.001**** | 0.032 | ***0.002**** | ***<0.001**** | 0.185 | 0.047 | 0.665 |
| LPA | 20.2  (19.1 – 24.3) | 14.7  (13.7 – 19.8) | 14.8  (7.0 – 17.5) | 8.1  (5.5 – 12.0) | ***<0.001**** | ***0.010**** | ***0.004**** | ***<0.001**** | 0.402 | 0.094 | 0.531 |
| MVPA | 1.6  (0.7 – 2.7) | 1.6  (0.8 – 3.2) | 0.1  (0.0 – 0.5) | 0.2  (0.0 – 0.4) | ***<0.001**** | 0.775 | ***0.002**** | ***<0.001**** | ***0.003**** | ***0.001**** | 0.965 |
| **Weekdays** | | | | | | | | | | | |
| SED | 36.8  (33.5 – 39.2) | 44.2  (36.2 – 47.0) | 43.3  (41.8 – 53.3) | 50.4  (47.4 – 54.5) | ***<0.001**** | 0.021 | ***0.002**** | ***<0.001**** | 0.257 | 0.052 | 0.565 |
| LPA | 21.0  (19.3 – 24.8) | 14.1  (12.3 – 20.2) | 16.6  (6.7 – 17.7) | 9.3  (5.5 – 12.5) | ***<0.001**** | ***0.010**** | ***0.003**** | ***<0.001**** | 0.386 | 0.080 | 0.509 |
| MVPA | 1.6  (0.8 – 2.5) | 1.4  (0.9 – 3.3) | 0.1  (0.0 – 0.7) | 0.2  (0.0 – 0.3) | ***<0.001**** | 0.978 | ***0.003**** | ***<0.001**** | ***0.008**** | ***0.001**** | 0.858 |
| **Weekend days** | | | | | | | | | | | |
| SED | 38.1  (33.9 - 42.1) | 40.5  (35.8 – 42.9) | 49.6  (42.7 – 52.7) | 54.1  (48.3 – 54.5) | ***0.001**** | 0.527 | ***0.009**** | ***<0.001**** | 0.059 | ***0.013**** | 0.740 |
| LPA | 20.7  (17.1 - 24.8) | 17.8  (16.5 – 20.3) | 10.3  (7.2 – 17.1) | 5.7  (5.1 – 11.7) | ***0.001**** | 0.266 | ***0.010**** | ***<0.001**** | 0.124 | 0.031 | 0.703 |
| MVPA | 0.7  (0.2 – 1.8) | 1.5  (0.4 – 3.5) | 0.1  (0.1 – 0.3) | 0.1  (0.0 – 0.6) | ***0.013**** | 0.251 | 0.039 | 0.048 | ***0.010**** | ***0.011**** | 0.781 |
| *Abbreviations*: TD, Typically Developing; CP, Cerebral Palsy; CP I-II, participants with CP classified in Gross Motor Functional Classification System (GMFCS) level I and II; CP III, participants with CP classified in GMFCS level III; CP IV-V, participants with CP classified in GMFCS level IV and V; IQR, Interquartile ranges; SED, sedentary; LPA, Low physical activity; MVPA, Moderate to vigorous physical activity. *Significant difference with *p*< 0.0167. | | | | | | | | | | | |

| Appendix IIIb: Overview of time (in minutes per hour) **adults** with cerebral palsy (CP) and typically developing (TD) peers spent in sedentary, low and middle to vigorous physical activity levels using cut-offs defined by **Evenson** and an indication of group differences. | | | | | | | | | | | |
| --- | --- | --- | --- | --- | --- | --- | --- | --- | --- | --- | --- |
| Parameters | TD  (n=30) | CP I-II  (n=10) | CP III  (n=5) | CP IV-V  (n=7) | *Overall Group effect* | TD vs  CP I-II | TD vs  CP III | TD vs  CP IV-V | CP I-II vs  CP III | CP I-II vs  CP IV-V | CP III vs  CP IV-V |
| (min/hour) | Median  [IQR] | Median  [IQR] | Median  [IQR] | Median  [IQR] | *p* | *p* | *p* | *p* | *p* | *p* | *p* |
| **Whole week** | | | | | | | | | | | |
| SED | 37.2  [34.9 - 40.0] | 43.6  [36.1 – 45.3] | 44.4  [42.5 – 53.1] | 51.5  [47.7 – 54.5] | ***<0.001**** | 0.034 | ***0.002**** | ***<0.001**** | 0.185 | 0.045 | 0.657 |
| LPA | 20.7  [19.5 - 24.6] | 14.9  13.9 – 20.6] | 15.1  [6.9 – 17.4] | 8.2  [5.5 – 12.1] | ***<0.001**** | ***0.014**** | ***0.003**** | ***<0.001**** | 0.320 | 0.079 | 0.584 |
| MVPA | 1.0  [0.4 – 1.9] | 1.2  [0.5 – 2.8] | 0.0  [0.0 – 0.3] | 0.2  [0.0 – 0.3] | ***<0.001**** | 0.606 | ***0.002**** | ***0.001**** | ***0.002**** | ***0.001**** | 0.843 |
| **Weekdays** | | | | | | | | | | | |
| SED | 37.0  [33.6 - 39.3] | 44.2  [36.2 – 47.0] | 43.4  [41.9 – 53.3] | 50.4  [47.5 – 54.5] | ***<0.001**** | 0.022 | ***0.002**** | ***<0.001**** | 0.255 | 0.051 | 0.565 |
| LPA | 21.3  [19.7 - 25.4] | 14.3  [12.6 – 21.0] | 16.6  [6.7 – 17.8] | 9.4  [5.5 – 12.5] | ***<0.001**** | ***0.011**** | ***0.003**** | ***<0.001**** | 0.335 | 0.072 | 0.541 |
| MVPA | 1.1  [0.5 – 1.8] | 1.3  [0.5 – 3.0] | 0.1  [0.0 – 04] | 0.1  [0.0 – 0.2] | ***<0.001**** | 0.751 | ***0.003**** | ***<0.001**** | ***0.005**** | ***0.001**** | 0.955 |
| **Weekend days** | | | | | | | | | | | |
| SED | 38.1  [33.9 – 42.2] | 40.6  [35.8 – 43.1] | 49.7  [42.8 – 52.8] | 54.3  [48.4 – 54.4] | ***0.001**** | 0.535 | ***0.009**** | ***0.001**** | 0.058 | ***0.012**** | 0.740 |
| LPA | 21.1  [17.2 – 24.8] | 18.5  [16.6 – 20.8] | 10.2  [7.2 – 17.1] | 5.5  [5.3 – 11.6] | ***0.001**** | 0.309 | ***0.009**** | ***<0.001**** | 0.106 | 0.024 | 0.702 |
| MVPA | 0.5  [0.1 – 1.0] | 1.2  [0.2 – 2.4] | 0.0  [0.0 - 0.2] | 0.1  [0.0 – 0.4] | ***0.010**** | 0.196 | 0.023 | 0.081 | ***0.004**** | ***0.015**** | 0.531 |
| *Abbreviations*: TD, Typically Developing; CP, Cerebral Palsy; CP I-II, participants with CP classified in Gross Motor Functional Classification System (GMFCS) level I and II; CP III, participants with CP classified in GMFCS level III; CP IV-V, participants with CP classified in GMFCS level IV and V; IQR, Interquartile ranges; SED, sedentary; LPA, Low physical activity; MVPA, Moderate to vigorous physical activity. *Significant difference with *p*< 0.0167. | | | | | | | | | | | |
